# Antibodies against PfEMP1, RIFIN, MSP3 and GLURP Are Acquired during Controlled *Plasmodium falciparum* Malaria Infections in Naïve Volunteers

**DOI:** 10.1371/journal.pone.0029025

**Published:** 2011-12-12

**Authors:** Louise Turner, Christian W. Wang, Thomas Lavstsen, Steven B. Mwakalinga, Robert W. Sauerwein, Cornelus C. Hermsen, Thor G. Theander

**Affiliations:** 1 Centre for Medical Parasitology at Department of International Health, Immunology, and Microbiology, University of Copenhagen and at Department of Infectious Diseases, Copenhagen University Hospital (Rigshospitalet), Copenhagen, Denmark; 2 Department of Medical Microbiology, Radboud University Nijmegen Medical Centre, Nijmegen, The Netherlands; Université Pierre et Marie Curie, France

## Abstract

Antibodies to polymorphic antigens expressed during the parasites erythrocytic stages are important mediators of protective immunity against *P. falciparum* malaria. Therefore, polymorphic blood stage antigens like MSP3, EBA-175 and GLURP and variant surface antigens PfEMP1 and RIFIN are considered vaccine candidates. However, to what extent these antibodies to blood stage antigens are acquired during naive individuals' first infections has not been studied in depth. Using plasma samples collected from controlled experimental *P. falciparum* infections we show that antibodies against variant surface antigens, PfEMP1 and RIFIN as well as MSP3 and GLURP, are acquired during a single short low density *P. falciparum* infection in non-immune individuals including strain transcendent PfEMP1 immune responses. These data indicate that the immunogenicity of the variant surface antigens is similar to the less diverse merozoite antigens. The acquisition of a broad and strain transcendent repertoire of PfEMP1 antibodies may reflect a parasite strategy of expressing most or all PfEMP1 variants at liver release optimizing the likelihood of survival and establishment of chronic infections in the new host.

## Introduction

Malaria caused by *Plasmodium falciparum* constitutes a major burden to large parts of the world despite efforts to reduce transmission and increase treatment. In malaria endemic populations, immunity to malaria is acquired slowly as a function of experienced infections. In regions with stable malaria transmission immunity to uncomplicated malaria is not acquired until adolescence [1] whereas protection against severe, non-cerebral malaria and death is obtained after only a few infections [2]. This development of immunity is thought to reflect the gradual acquisition of effective cells and antibodies directed against malaria polymorphic and variable *P. falciparum* blood stage antigens [3]–[6]. Identification of these effective antibodies may lead to a vaccine mimicking the natural acquired protection against malaria. Identified polymorphic blood stage vaccine candidates include merozoite surface protein 3 (MSP3), erythrocyte-binding antigen 175 (EBA-175) and glutamate-rich protein (GLURP). MSP3 and EBA-175 are located on the extracellular merozoite surface and are involved in red blood cell invasion [7], [8] while GLURP is expressed in both the pre-erythrocytic and erythrocytic stage [9] but no function has yet been ascribed. Of considerable interest are also the variant surface antigens *Plasmodium falciparum* erythrocyte membrane protein 1 (PfEMP1) and RIFINs. PfEMP1 mediate adhesion to human endothelial receptors [5], [10]–[13] probably to avoid clearance by the spleen [14] whereas the function of RIFINs is yet unknown, although it is proposed that they expose their highly polymorphic V2 region on the surface of infected erythrocytes and therefore contributing to the antigenic variation of a *P. falciparum* infection [15], [16]. PfEMP1 molecules are encoded by a repertoire of around 60 different *var* genes per genome but are generally thoutht to be expressed one at a time [17], [18], although the co-expression of two different PfEMP1 variants has been observed in the laboratory-cultured parasite clone 3D7 [19]. The extracellular variable part of PfEMP1 contains an N-terminal segment followed by segments composed of two main domain types, Duffy binding-like domains (DBL) and cysteine-rich inter-domain regions (CIDR), which can be further divided into classes and sub-classes based on sequence similarity [20]. The *var* genes are divided into four main groups (A, B, C and VAR2CSA), each group shares specific 5′ promoter regions and phylogenetically distinct DBL and CIDR domains [20], [21], [22]. The VAR2CSA PfEMP1 is involved in pregnancy malaria by facilitating parasite sequestration in the placenta [13]. Moreover, immunological studies imply that an antigenically conserved subset of PfEMP1 are associated with severe disease in children [23], [24], [25]. Recently, it was shown that children living in areas of high *P. falciparum* transmission gradually but most rapidly acquire a broad anti-PfEMP1 antibody repertoire and antibodies against DBL domains of the group A PfEMP1 variants are acquired first [26]. Group A PfEMP1 have previously been associated with severe childhood malaria by studies of both *var* expression and PfEMP1 antibody acquisition [27]–[31]. Studies of *var* expression in controlled experimental infections of naïve Dutch individuals infected with the NF54 parasite strain (parental strain of the 3D7 clone) have suggested that most of the different parasites released from the liver cells express different *var* genes and that continuous growth may favour parasite expressing PfEMP1 variants facilitating the most effective sequestration to host endothelium [32]. However, it has not been known if the short period of infection (1–5 days or 1–3 post liver release parasite life cycles) in experimentally infected volunteers and the parasite densities obtained (low maximum parasitaemia <44.000 parasites/ml) are sufficient to induce antibody responses to the above mentioned bloodstage antigens. Therefore, plasma samples collected from controlled malaria infections of naïve volunteers [33]–[37] were used to investigate the acquisition of antibodies to an array of 104 PfEMP1 domains, eight RIFINs, MSP3, EBA-175, and GLURP.

## Materials and Methods

### Ethics statement

Informed consent form was signed by all subjects and the trials were reviewed and approved by the Ethical Committee of the Radboud University Nijmegen Medical Center (CWOM: 0004-0090, 0011-0262, 2001/203, 2002/170, and 2004/129) and the Central Committee for Research Involving Human Subjects of The Netherlands (CCMO NL24193.091.09) as previously described [33]–[38].

### Experimental infections of human volunteers and plasma samples

This study benefits from the plasma collected from naïve volunteers infected with the *Plasmodium falciparum* NF54 isolate by bites of *Anopheles stephensi* mosquitoes, and treated after a few rounds of asexual parasite multiplication, when parasites could be detected or the patient developed symptoms [33]–[37]. These controlled human malaria infection (CHMI) experiments were conducted in six series, between which the experimental set up (number of infectious mosquito bites, day of treatment relative to first detection of asexual parasitemia differed slightly (Table S1). From these 44 volunteers blood samples were taken on the day before infection and on day 21, day 21 and 42, day 21 and 90, day 35, day 42, or day 90 post infection from 14, 11, 9, 5, 1, and 4 volunteers, respectively (Table S1). In addition, we analysed plasma from 10 volunteers who had been immunized by receiving three rounds of experimental infections under a chloroquine prophylaxis and then challenged by a fourth exposure to infectious mosquito bites without chloroquine cover [37]. These individuals experienced brief asymptomatic very low density asexual infections during the immunisation phase, but no asexual parasitemia were detected during the challenge [37]. From these individuals plasma collected the day before exposure to the bites of the infectious mosquitoes (days 0, 32, 60, 116 after the initiation of the experiment)and on days 35 and 140 after the challenge infection (days 151 and 256 after initiation of the experiment) were available (Table S1). Plasma samples were stored at −80°C.

### Protein expression

Protein expression was as previously described [27], [39]. In short, primer pairs designed to contain restriction enzyme sites were used to amplify *var* gene fragments encoding 104 PfEMP1 domains (67 DBL, 32 CIDR, 5 multi-domain constructs) representing four different genetic backgrounds (see Table S2): 3D7/NF54: 39 DBL, 15 CIDR, 3 DBL-DBL and 2 DBL-CIDR; HB3: 13 DBL and 8 CIDR; IT/FCR3: 15 DBL and 8 CIDR; Dd2: 1 CIDR domain. Primer pairs amplifying eight RIFIN V2 domains and the F2 region of EBA-175 from 3D7/NF54 genomic DNA were also used (Table S2). The R0, R1, R2 (N-terminal non-repeat, central repeat and C-terminal repeat regions of GLURP), and the C-terminal region of MSP3 were included in the study as well [40], [41]. Digested PCR products were cloned into the *Baculovirus* vector, pAcGP67-A (BD Bioscience) designed to contain the V5 epitope upstream of a histidine tag in the C-terminal end of the constructs or in the case of the RIFINs a GST tag. Identity of the cloned fragments was verified by sequencing. Linearized Bakpak6 Baculovirus DNA (BD Biosciences Clontech) was co-transfected with pAcGP67-A into Sf9 insect cells for generation of recombinant virus particles and histidine-tagged proteins secreted into the supernatant of infected High-Five insect cell were purified on Co2+ metal-chelate agarose columns. Eluted products were dialysed overnight in PBS. The yield, integrity and purity of the recombinant proteins were estimated by analysis on SDS gel, comparing to BSA standards, and by western blotting using the anti-V5 Ab. All of the proteins coupled to the Luminex beads were estimated to be at or above 80% purity.

### Covalent coupling of recombinant PfEMP1 proteins to beads

Carboxylated Luminex microspheres were covalently coated with the different protein domains through an interaction of their carboxyl groups and the amino groups on the proteins following the procedure suggested by the manufacturer. Microspheres (1.25×10^7^ microspheres/ml) were brought to room temperature, vortexed for 1 min, and transferred to Eppendorf tubes. The supernatant was removed after centrifugation for 1 min at 16,000 x g. One millilitre of distilled water was added to the microspheres, vortexed for re-suspension, followed by centrifugation for 1 min at 16,000 x g. The microspheres were sonicated in a water bath sonicator into suspension and centrifuged for 1 min at 16,000 x g. The supernatant was removed and 1 ml of activation buffer (0.1 M NaH2PO4 (pH 6.2)) was added to the pellet and vortexed for re-suspension. In separate tubes 1-ethyl-3-(3-dimethylaminopropyl)-carbodiimide hydrochloride (EDC) and N-hydroxysulfosuccinimide (Sulfo-NHS; Pierce Biotechnology) were reconstituted to 50 mg/ml, and 125 µl of each was added to the microspheres, vortexed, and incubated at room temperature for 20 min with inversions in the dark. The microspheres were centrifuged for 1 min at 16,000 x g, re-suspended in 1 ml of 50 mM MES (pH 5.0), centrifuged for 1 min at 16,000 x g, and the supernatant was removed. The MES wash was repeated. The microspheres were re-suspended in 500 µl of MES. In separate tubes, the different protein samples (100 µg per ml of microspheres) were mixed with MES to a final volume of 500 µl and each was added to a separate microsphere population and incubated at room temperature for 2 h in the dark with inversions. The microspheres were centrifuged for 1 min at 16,000 x g and the supernatant was removed. The microspheres were washed twice in 1 ml of PBS/TBN (0.02% Tween 20, 0.1% BSA, and 0.05% sodium azide in PBS (pH 7.4)). The microspheres were re-suspended in 1 ml of PBS/TBN and stored at 4°C in the dark. To determine whether coupling was effective, aliquots of the different microsphere sets were prepared for analysis as described below and analyzed on the Luminex instrument.

### Multiplexing and lyophilization of microspheres

Equal volumes of the coated microspheres were pooled together and mixed by vortexing. Sucrose and Tween 20 were added to 3% and 0.05%, respectively, mixed by vortexing, and single-use aliquots were lyophilized (AdVantage, Wizard 2.0; VirTis) in polypropylene vials, sealed under nitrogen gas, and stored at −80°C. Immediately before use, lyophilized microspheres were reconstituted with distilled water and used for analysis [43] as described below.

### Analysis of coupled microspheres on the Luminex

The coated microspheres were diluted 1/333 in assay buffer E (ABE buffer, 0.1% BSA, 0.05% Tween 20, 0.05% sodium azide in PBS (pH 7.4)) and 50-µl aliquots were dispensed into the wells of a 1.2-µm filter bottom 96-well microtiter plate (MSBVS 1210; Millipore) pre-wetted with ABE buffer. The microspheres in 96-well plates were washed three times with ABE using a vacuum manifold (Millipore). Frozen plasma samples were thawed at room temperature, mixed by vortexing, and spun at 16,000 x g for 5 min to remove particulates. Plasma samples were diluted 1/80 in ABE buffer and 50-µl aliquots of diluted sample were added to the microspheres and incubated in the dark on a shaking platform at 1100 rpm for 30 s followed by 300 rpm for 30 min. Excess antibody was removed using a vacuum manifold followed by three washes in ABE. 25 µl of biotinylated human IgG (Sigma-Aldrich) detection antibody diluted 1/500 in ABE was added to the microspheres, incubated in the dark with shaking at 1100 rpm for 30 s, followed by 300 rpm for 30 min and washed three times in ABE. 50 µl of streptavidin-PE (Sigma-Aldrich) diluted 1/500 in ABE was added to the microspheres and incubated in the dark with shaking at 1100 rpm for 30 s, followed by 300 rpm for 10 min. Excess streptavidin-PE was removed followed by three washes in ABE. The microspheres were then re-suspended in 125 µl of ABE and analyzed on the Luminex instrument. The reader was set to read a minimum of 100 microspheres per microsphere region and results were expressed as median fluorescent intensity.

### Statistical analyses

The purpose of the study was to determine to which degree the volunteer's acquired antibodies to the tested malaria antigens. To this end the reactivity (measured in MFI) in the sample collected from each individual prior to malaria exposure was used as a baseline. Individuals were defined as having acquired an IgG response if the MFI in the sample collected during the experiment/MFI in the sample collected at the beginning of the experiment was >3 and the MFI in the sample collected during the experiment was >500. This conservative cut-off definition was used to assure that responder status not reflected general increases in IgG levels or small variations in background levels. To compare acquisition of antibodies between those who were experimentally infected and those who were protected by repeated immunisations before challenge we used Wilcoxon rank-sum test for unpaired data to compare the number of PfEMP1 domains to which antibodies were acquired. Relations between acquisition of PfEMP1 antibodies and parasite load, maximum parasitemia, number of mosquito bites and number of asexual cycles completed before treatment was done using Spearman's rank sum test. Stata 12 (http://www.stata.com/stata12/) was used for the analyses.

## Results

### Acquisition of IgG to GLURP, MSP3, and EBA-175

**Figure 1:**
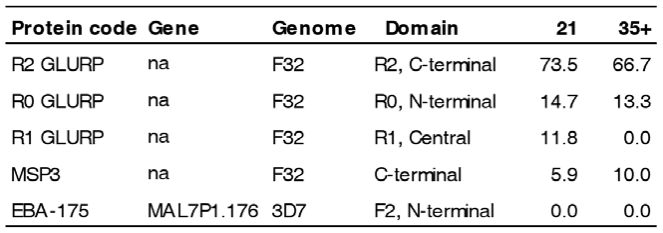
Proportion of malaria naïve volunteers acquiring IgG against GLURP, MSP3 and EBA175 antigens after controlled experimental *P. falciparum* infections. The percentage of malaria infected volunteers with a measurable IgG response against GLURP R0, R1, R2, MSP3 and EBA175 malaria antigens on day 21 (N = 34) and/or day 35, 42 or 90 (N = 30) post infection in descending order. In total 44 volunteers were included (Table S1) and the IgG response was measured by bead-based Luminex technology.

Antibody levels to N-terminal, Central and C-terminal repeat regions (R0, R1 and R2) of GLURP, C-terminal region of MSP3 and the N-terminal F2 region of EBA-175 were measured in plasma collected from 44 naïve Dutch volunteers before and after a controlled human malaria infection with *Plasmodium falciparum* isolate NF54 which is a relatively short infection period. Around 70% of the volunteers acquired IgG against R2 whereas IgG to R0 and R1 only were acquired by approximately 13% (Figure 1). IgG with specificity to MSP3 was acquired by ∼ 10%. None of the volunteers acquired IgG antibodies to EBA-175 (Figure 1).

### Acquisition of anti-PfEMP1and anti-RIFIN IgG

**Figure 2:**
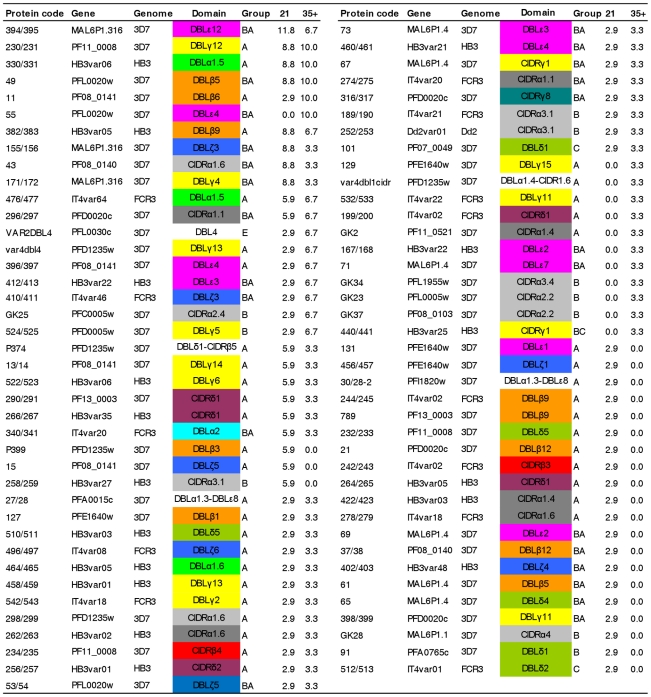
Proportion of malaria naïve volunteers acquiring IgG against PfEMP1 antigens after controlled experimental *P. falciparum* infections. The percentage of malaria infected volunteers with a measurable IgG response against 79 PfEMP1 malaria antigens on day 21 (N = 34) and/or day 35, 42 or 90 (N = 30) post infection in descending order. The PfEMP1 domain classification was according to Rask et al [19]. 25 DBL and CIDR domains were omitted from the figure as there was no IgG recognition of these domains (see Table S2). In total 44 volunteers were included (Table S1) and the IgG response was measured by bead-based Luminex technology.

**Figure 3:**
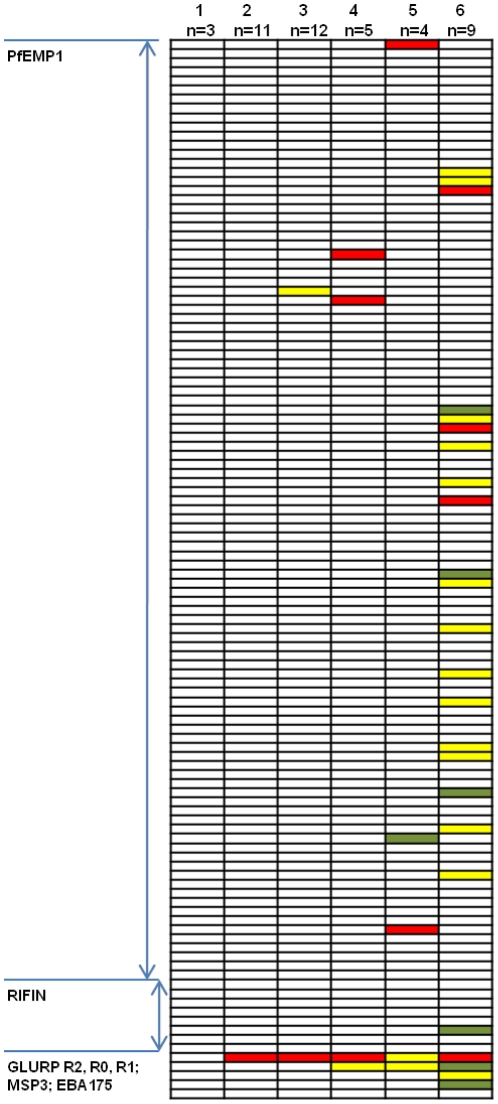
Acquisition of IgG to 104 PfEMP1 domains, eight RIFINs, and GLURP, MSP3, and EBA-175 antigens of *Plasmodium falciparum* in malaria naïve individuals exposed to controlled experimental *P. falciparum* infections. The IgG reactivity pattern of the six groups represented by their acquisition pattern of IgG to blood stage antigens observed in the 44 examined volunteers after exposure of a short *P. falciparum* infection: Volunteers with no acquisition of antibodies to any of the tested malaria antigens (*n* = 3); volunteers who acquired antibodies to GLURP only (*n* = 11); and volunteers who acquired antibodies to 1, 2, 3 or >3 PfEMP1 domains, respectively, as well as one or more merozoite antigens (*n = *12, 5, 4 and 9). Results are expressed as median fluorescent intensity (MFI): Red >2000 MFI; Green >1000 MFI; Yellow >500 MFI (cut-off value).

**Figure 4:**
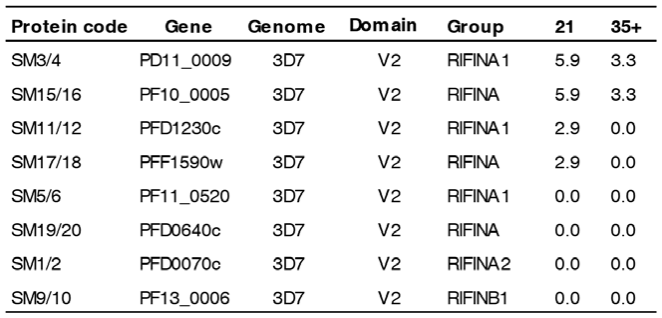
Proportion of malaria naïve volunteers acquiring IgG against RIFIN antigens after controlled experimental *P. falciparum* infections. The percentage of malaria infected volunteers with a measurable IgG response against eight RIFIN malaria antigens on day 21 (N = 34) and/or day 35, 42 or 90 (N = 30) post infection in descending order. The grouping of RIFINs was according to Wang et al. [45]. V2: Variable domain 2, the putative extracellular polymorphic region defined by Cheng et al. [15]. In total 44 volunteers were included (Table S1) and the IgG response was measured by bead-based Luminex technology.

IgG reactivity to 104 recombinant PfEMP1 domains amplified from 3D7/NF54, HB3, IT/FCR3 and Dd2 genomes were measured in plasma samples from the same 44 individuals before and after *P. falciparium* infection. Of those 104 domains, 79 were recognized by between 3 and 12% of the volunteers (Figure 2) with no clear distinction in the recognition of domains according to domain subclass or PfEMP1 group. The pattern of antibody acquisition varied considerably among the 44 volunteers, but could be divided into six groups represented by their acquisition pattern (Figure 3). The 44 volunteers were grouped into individuals who did not acquire antibodies to any of the malaria antigens (*n = *3; 7%), individuals who acquired antibodies to GLURP only (*n = *11; 25%), and individuals who acquired antibodies to 1, 2, 3 or >3 PfEMP1 domains as well as to one or more of the merozoite antigens (*n = *12, 5, 4 and 9; in total ∼68%). 41% of the 44 volunteers responded to a non-NF54 PfEMP1 domains and of the 45 PfEMP1 domains amplified from *P. falciparum* HB3, IT/FCR3 and Dd2 genomic DNA, 31 were recognized (Figure 2). The pattern of antibody acquisition did not correlate with the parasite load, maximum parasitemia, number of mosquito bites or number of intra-erythrocytic parasite cycles. Antibody levels to the extracellular polymorphic domain [15], V2, of eight recombinant RIFIN proteins were also tested. Six volunteer acquired antibodies to in total four of the eight RIFINs (Figure 4).

### Acquisition of antibodies to blood stage antigens during an immunization study

**Figure 5:**
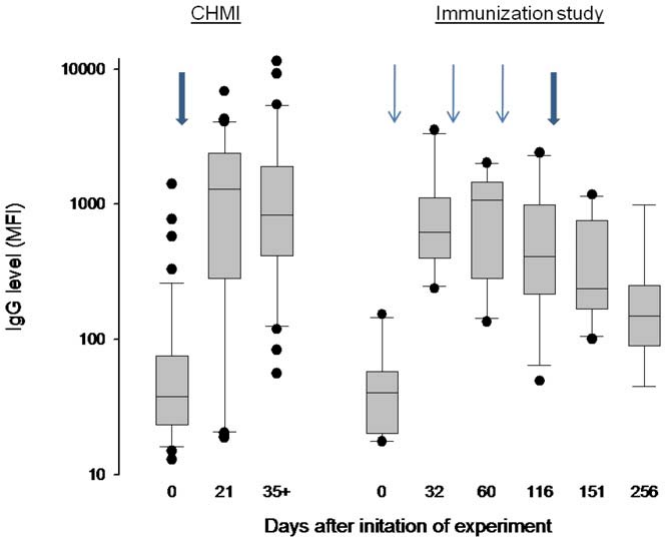
Acquisition of IgG to GLURP R2 during a liver stage immunization study. The anti-GLURP R2 IgG levels in 44 volunteers experimentally infected with *Plasmodium falciparum* (indicated by a thick arrow day one) and in 10 volunteers immunized by three exposures to *P. falciparum* while treated with chloroquine killing blood stage parasites (indicated by thin blue arrows day one, 33 and 61) and after challenge without a drug cover (thick blue arrow day 118). “35+” represents plasma samples taken 35, 42 or 90 days after infection. CHMI: controlled human malaria infection.

**Figure 6:**
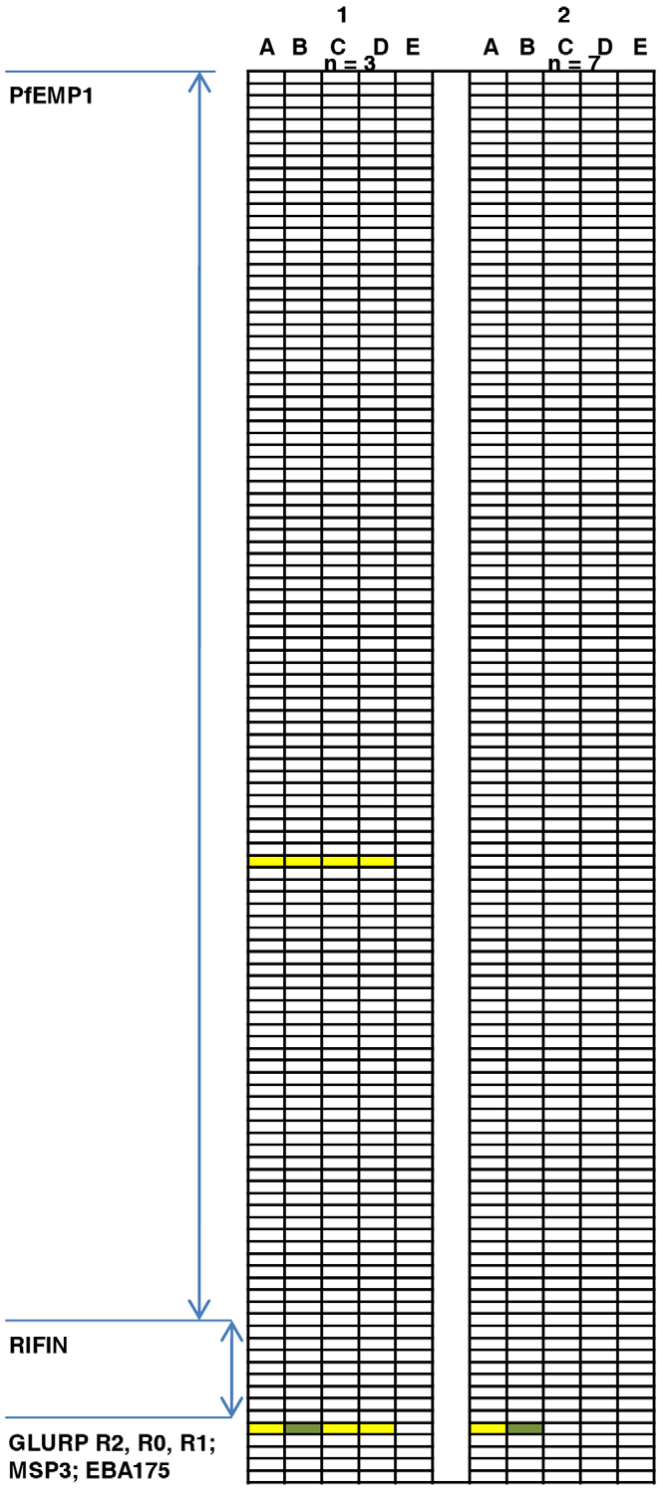
Acquisition of IgG Abs to 104 PfEMP1 domains, eight RIFINs, and GLURP, MSP3, and EBA-175 antigens in two immunized volunteers. The two volunteers, 1 and 2, were selected to represent volunteers with acquisition of antibodies to PfEMP1 and merozoite antigens (*n* = 3) and volunteers with acquisition of antibodies to the merozoite antigens only (*n* = 7). The letters A, B, C, D and E correspond to 32, 60, 116, 151 and 256 days after first immunization. Parasite challenges were at day 118 [37]. The results are expressed as median fluorescent intensity (MFI): Red >2000 MFI; Green > 1000 MFI; Yellow > 500 MFI (cut-off value).

During an immunization study at Radboud University, Nijmegen [37], 10 volunteers were immunized by NF54 parasites by the bites of infectious mosquitoes three times with an interval of one month and concurrently receiving chloroquine, a drug that kills blood stage parasites. One month after discontinuation of the drug, when chloroquine levels were below therapeutic concentrations, the volunteers were challenged with NF54 by mosquito bites. A progressively reduced incidence and burden of blood stage parasitemia was measured by PCR during the immunization phase while there was no evidence of blood stage infection during the challenge phase [37]. These individuals acquired anti-GLURP R2 IgG after the first immunization and the antibody levels decreased after the third immunization. The peak anti-R2 IgG levels in the immunized group were comparable to the peak levels among the 44 individuals exposed to one infection (Figure 5). By contrast the acquisition of anti-PfEMP1 IgG was lower among the immunized individuals than among the individuals exposed to one infection. The immunized individuals acquired antibodies to 0 [0;2] (median and 95% confidence interval, CI) domains. Among the 44 individuals exposed to one infection the number was 1 [CI 0;19] (*P* = 0.02 compared to the immunized individuals). Among the five individuals serving as controls in the immunization study the number was 1 [0;2] (*P* = 0.10 compared to the immunized individuals). Seven of the 10 immunized volunteers did not acquire IgG against any of the PfEMP1 domains. Figure 6 shows the reactivity pattern in one of the three volunteers who responded to PfEMP1 during the study and one who did not. Among the immunized individuals one acquired IgG to a single RIFIN and none acquired IgG to the GLURP R0, GLURP R1, MSP3 or EBA-175 domains.

## Discussion

This study was conducted to assess the immune response to PfEMP1 in experimentally infected individuals. Malaria naïve individuals were infected with *P. falciparum* after bites by mosquitoes and the infection progressed through the liver, but was terminated by drug treatment of blood stage parasites after a few asexual multiplication rounds. Since the PfEMP1 repertoire of the NF54 parasites used to infect the volunteers is known, and we had access to recombinant PfEMP1 domains made on basis of NF54 sequences and domains made on the basis of PfEMP1s from other parasites, we could assess the acquisition of antibodies recognizing homologues sequences as well as acquisition of cross reactive antibodies. Despite the short exposure to asexual blood stage parasites two thirds of the 44 volunteers acquired PfEMP1 antibodies. The breadth of antibody response varied considerably between the volunteers. Some only acquired antibodies to one of the 104 PfEMP1 domains tested while one volunteer acquired antibodies to 38 domains. We could not establish any association between the course of infection in the volunteers (parasite load, maximum parasitemia, number of asexual multiplication cycles) and the breadth of the PfEMP1 antibody response. Thus the variation between the individuals may reflect individual differences in the regulation and induction of immune response between the individuals or stochastic variation. PfEMP1 can be divided into group A-C and VAR2CSA. Antibodies were acquired to domains present in all groups without any particular order or pattern. This is in agreement with the hypothesis supported by *var* transcript analyses in the same volunteers, that all or most variants are expressed by the parasite population at the onset of the blood stage infection to maximize the survival in a new host with unknown immune status [32], [46]. The same *var* transcript analyses also provided the only formal evidence in support for the hypothesis that parasites that express a subset of PfEMP1, potentially due to better cytoadhesion properties, bestow higher parasite growth rates in naïve individuals. The PfEMP1 antibody acquisition data presented here could not verify this hypothesis, probably because antibody measurements detect the accumulated acquired antibody repertoire and not directly reflects parasite densities. In 2007, Elliot et al [47] showed that individuals who had experienced brief infections during travels in Africa, Asia or the Western Pacific had acquired IgG recognizing variant surface antigens (VSA) on six different *P. falciparum* lines, indicating that IgG against VSA are broadly cross reactive. In our study, individuals were exposed to the defined repertoire of PFEMP1 present in NF54. Interestingly, 41% of the volunteers acquired IgG to non-NF54 PFEMP1 domains and two thirds of the 45 non-NF54 PfEMP1 domains were recognized by at least one volunteer. We have previously shown that there is little cross reactivity between antibodies directed against PfEMP1 domains produced on a 3D7 genetic background [48]. The present study indicates that even short exposure to a particular PfEMP1 domain can induce antibodies which react with PfEMP1 domains encoded from different genomes. Combined the studies suggest that there is little serological cross reactivity between intra-genomically encoded PfEMP1 and broad cross reactivity between inter-genomically encoded PfEMP1. This cross reactive PfEMP1 immune response, which cannot in these experiments be explained by a sequential exposure to PfEMP1 epitopes, could explain why children in endemic areas relatively quickly generate a broad repertoire of anti-PfEMP1 antibodies, [26], [49]. Such an antibody response may also enhance the parasites likelihood of establishing a chronic infection [50].

We also assessed the acquisition of antibodies to selected merozoite antigens and RIFINs. Two thirds of the volunteers acquired antibodies to the C-terminal R2 repeat region of GLURP, while only between 0–15% acquired antibodies to GLURP R0, GLURP R1, MSP3, EBA-175 and the RIFINs. This is in agreement with a previous observation where no antibodies were found against a GLURP_85–213_ long synthetic peptide which did not include the R2-region [37]. The high recognition of GLURP R2 could reflect that this region is particularly immunogenic [51] and/or that the antibodies are induced by liver parasites expressing GLURP [9]. It has previously been reported that antibodies to GLURP R2 and other malaria antigens can be induced by transient exposure to *P. falciparum* [52]–[55].

We also investigated the antibody response in individuals who were immunized by three exposures to infected mosquito bites while under chloroquine treatment. Surprisingly, these immunized individuals were protected, when they receive a fourth exposure to infected mosquitoes in the absence of chloroquine [37]. During immunization, the attenuated infections gave raise to brief very low density blood stage parasiteamia. The question was whether these brief episodes were sufficient to induce anti-PfEMP1 antibodies, which could mediate protection during challenge. Our data do not support this hypothesis, since only a few of the immunized individuals acquired anti-PfEMP1 antibodies to a few of the domains. Similarly, the immunized individuals did not acquire antibodies to the most of the other blood stage antigens tested. The exception was anti-GLURP R2 antibodies, which were acquired after the first parasite exposure in the immunized individuals at levels that were similar to those measured in the non-immunized volunteers. In the immunized individuals, these antibodies could have been induced by a brief exposure to low levels of asexual parasites or induced by GLURP or other glutamine rich proteins expressed by parasites stages in the pre-erythrocytic life cycle [9].

In conclusion, PfEMP1, RIFIN, GLURP and MSP3 antibodies are acquired after short controlled *P. falciarum* infections suggesting that the immunogenicity of the variant surface antigens is similar to the less diverse merozoite antigens and broad and strain transcendent PfEMP1 reactivity may reflect a parasite strategy of expressing most or all PfEMP1 variants at liver release optimizing the likelihood of survival and establishment of chronic infections in the new host.

## Supporting Information

Table S1 **Overview of the 54 volunteers involved.**
(XLS)

Table S2 **Primers used for** ***Plasmodium falciparum*** **antigen expression.**
(XLS)
